# Valyl

**Published:** 1905-11

**Authors:** A. Ammelburg


### Valyl.

BY A. AMMELBURG.

Valerian preparations have been, since olden times, highly esteemed by physicians. While these preparations, however, were formerly utilized for the treatment of all possible ailments, in recent years the preference for them by physicians has been visibly lessened. The aimed-for results were not positive; their effect was not to be depended on. As Kochman (1) has shown, the grounds for fluctuations in their efficacy lay in the marked liability to disintegration of these valerian preparations. Infusions, even when freshly prepared, as well as the different tinctures, dialysates and extracts of the valerian root, after a short time, lose their original neutral reaction and become acid, as has been established by titration.

What does this appearance of acid reaction signify? The bearer of the efficacy of valerian root is the ethereal oil, and in this again there is, as Kionka (2) has shown, the ester of the valerian acids to be denoted as the components whose action comes nearest to the specific valerian effect. Such esters of the fatty acid series are, however, as has been long known, very easily oxidized into the respective acids by splitting up, and even the presence of water alone can bring about this hydrolytic division. The fatty acids arising thereby, namely, the valerianic acid, are, however, as we know from the examination of Harras (3), pharmacodynamically inactive. The acidification of the valerian preparations thus goes hand in hand with an impairment of its therapeutical value. The wish is, therefore, justifiable that instead of the old, unstable and in their effect, inconstant valerian preparations, to introduce substitute remedies which do not decompose so easily, and thus become inactive.

The esters of the valerian acids, as stated above, are preserved in the dry drug itself and possess a certain valerian effect, but are not appropriate for use on account of their easy disintegration. They are decomposed hydrolytically by water alone. The introduction of the valerianicacidborynlester should, therefore, be regarded as a mistake.

From validol, too, which is half valerianicacidmethyl ester, we

*Translated for THE TEXAS MEDICAL JOURNAL from *Aertzlichen Central Anzeiger* No. 16, April 17, 1905

should expect but little valerian effect. In this preparation there arises essentially another component to action—the menthol.

There is, however, another series of derivatives of the valerianic acid which also presents the picture of valerian action, but not so easily decomposed. These are the amids of the valerianic acids. Among these, according to the researches of Kionka and Liebrecht (4), valerianicacid-diethylamid has proven itself by far the most effective, and has, therefore been introduced into practice under the name of *Valyl.*

According to Kionka (5), the following effects are regarded as specific valerian effects:

1. An exciting action on the mind (Psyche).

2. In small doses, an exciting action on the central nervous system.

3. After large doses, a central motor and sensory palsy and abolition of the reflex activity. The latter may also occur occasionally after small doses.

4. In small doses, an increased blood pressure effect, produced on the one hand by an action on the vasomotor (narrowing of the periphery vessels), and on the other by an exciting effect on the heart's action itself.

5. In large doses, a diminished blood pressure effect produced by vasomotor palsy and direct injury of the heart.

6. Even after small doses, short lasting fall of the blood pressure at regular intervals. This is produced by momentary dilatation of the peripheral vessels.

Valyl possesses all of these effects in a high degree. It has abundantly proven itself therapeutically practicable, and has been meritoriously commended from different directions.

Its main indication, naturally, like all valerian preparations, is in hysteria with its manifold symptoms. Cases of severe grade, also hysteria virilis, invariably respond promptly to Valyl, as was proven by the first examinations of the preparation conducted at the Breslan University clinics and hospitals.

No less favorable for use are cases of neurasthenia and hypochondria as well as neuroses and certain forms of neuralgia, as has been proven by the researches of Bardet (6), Meyer (7), and Goldman (8) and others. Particularly favorable for Valyl therapy to which Klemperer (9) has called attention, is the cardiac neuroses, and other pure nervous heart complaints. Just as valerian tincture is often utilized by nervous patients as a house remedy to secure the necessary rest in sleep, this object is reached with greater certainty by the administration of Valyl.

Finally, there are of nervous ailments for which Valyl treatment is suitable yet to mention: certain forms of neuralgia and hemicrania. Severe cases of movable darkening in the field of vision of long duration were also promptly cured by Valyl.

A still further extensive field for the application of Valyl is the manifold disturbances of menstruation and the ailments occurring during the menopause and during pregnancy. Fritsch (10) and especially Frendenberg (11) have recognized the high value of this medication. In disorders of menstruation, the congestion and the pains in the lower abdomen are relieved by Valyl, also the existing headaches, and, among other things, it diminishes the profuse hemorrhage. The hot flushes, agitation, cardiac palpitation, which mainly appear at the change of life, are also relieved in patients with normal menstruation.

The fifth effect, the fall of blood pressure resulting after large doses, has been therapeutically utilized by Alter (12). He employed Valyl to combat the state of agitation in periodical mania. Whereas, these conditions, as was revealed by tonograph measurements, go regularly with a raising of the blood pressure, Alter gave in such cases rapidly increasing doses of Valyl. He secured thereby, as was shown through examination by means of the tonograph, a gradual developing but sufficient fall of the blood pressure, while at the same time the psychical excitement disappeared.

Valyl is given only in gelatin capsules of 0.125 gram, mixed with an equal part of cetaceum. Not only is an unlimited stability of the Valyl thereby guaranteed, but the disagreeable taste avoided. Valyl, like all other fatty acid amides as the ester of fatty acids (bornyval) and the camphor kind (menthol, validol) possesses, when applied the substance to sensitive mucous membranes, a peculiarally local irritating effect which, it is true, provokes no material harm to the mucous membrane (inflammation) but causes a disagreeable burning sensation. For this reason, all these substances should be given in capsule form. Valyl capsules should not be taken on an empty stomach, but during or directly after a meal, followed by milk or soup, etc.

The general dose is two or three capsules two or three times daily. However, one may without hesitation, administer a greater number. Thus, Alter, to secure an energetic fall of blood pressure, gave ten capsules three times daily. He gave this dose for several days without harm. Ordinarily two or three capsules two to three times a day will be found sufficient.

In treating the various ailments, it is best to begin with the average dose, and then according to the symptoms increase or decrease the dose.

As a rule, nervous insomnia is successfully treated with two or three capsules, taken with a glass of milk upon going to bed.

In the treatment of non-periodical agitation and other ailments of the climacteria and pregnancy, one must adapt the treatment to the individual behavior of the patient.

In dysmenorrhea, the following directions are recommended: About three days before the beginning of the expected menstruation, administer one or two capsules of Valyl three times daily. As soon as menstruation is established, increase at once to three or four capsules three times daily, and only after a decreased flow, reduce gradually to three capsules a day, and continue for three or four days more. In the interval between the menstruation it is desirable to continue Valyl treatment.

---

1. M. Kochmann: Ueber die Veranderlichkeit der Baldrian-preparat Deutsche med. Wochenschr., 1904, No. 2.

2. H. Kionka: Die Wirkung des Baldrians-Intern. Archiv. f. Pharmakodynamie n. Therapie. Bd. XIII, p. 215.

3. Harras: Ebendort. Bd. XI, p. 431.

4. Kionka and Liebrecht: Ueber ein neues Baldrianpraparat (Valeriansaurediaethylamid) Deutsche med Wochensch.rift, 1901, No. 49.

5. Kionka: l. c.

6. Bardet: Quelques considerations sur un nouvel Antinervin-Bullet. gen. d. Therapeutic. May 15, 1903.

7. Meyer: Un nuovo preparats di Baldriana; il Valyl. Gazzetta intern. di. Medicini, 1903, No. 23.

8. Goldman: Ueber Valyl. Hellmittel Revue, 1904, No. 1.

9. G. Klemperer: Valyl, ein empfehlenswertes Baldrianpraparat-Therapie d. Gegenw. January, 1902.

10. Fritsch: Klimakterische Beschwerden, Die deutsche Klinik am Eingang des XX Jahrhunderts. Bd. 9, p. 575.

11. Freudenberg: Valyl—Der Frauenarzt, May 16, 1902.

12. Alter: Valyl—Therapie d. Gegenw. 1904, No. 3.

